# A study to investigate the relationship between transcranial magnetic stimulation on cognitive impairment and neurotrophic factor in post-stroke patients

**DOI:** 10.3389/fneur.2025.1603870

**Published:** 2025-08-29

**Authors:** Wenyan Li, Yinghua Wen, Wei Li, Jingjing Liu, Sha Liu, Junying Wu, Yao Gao, Yong Xu

**Affiliations:** Department of Rehabilitation Medicine, The First Hospital of Shanxi Medical University, Taiyuan, China

**Keywords:** transcranial magnetic stimulation, stroke, brain-derived neurotrophic factor, nerve growth factor, working memory, post-stroke cognitive impairment

## Abstract

**Objective:**

This study aimed to investigate the efficacy of transcranial magnetic stimulation on post-stroke patients in terms of cognitive impairment, and to observe its relationship with peripheral blood neurotrophic factor concentration and changes in brain area function.

**Methods:**

Sixty patients with cognitive impairment after ischemic stroke were randomly assigned to group A (*n* = 30) and group B (*n* = 30) to receive TMS and sham stimulation of the left dorsolateral prefrontal cortex, respectively. The frequency of magnetic stimulation intensity in the TMS group was 10 Hz, and 10 stimulations were applied in the left DLPFC. Montreal Cognitive Assessment (MoCA), Digital Breadth Test (DST) and N-Back reaction times as well as determination of peripheral blood BDNF, NGF concentrations were assessed before and 2 weeks after stimulation, respectively, and the functional connectivity of each brain region in the assessment task state was analyzed using near infrared spectroscopy (fNIRS). Finally, a correlation study between peripheral neurotrophic factors and brain regions and in relation to cognitive scales was performed.

**Results:**

After stimulation, patients in the TMS group had increased MoCA (*p* = 0.026), DST (*p* = 0.008) and N-Back (*p* = 0.007) scores compared to the sham stimulation group, as well as increased peripheral blood BDNF (*t* = 2.448, *p* = 0.021) and NGF (*t* = 2.885, *p* = 0.007) concentrations. The Pearson’s correlation interaction effect was significant between the patients’ left DLPFC brain region and the right DLPFC brain region (*r* = 0.492, *p* = 0.038). BDNF was negatively correlated with the N-Back (*r* = −0.4668, *p* = 0.038), NGF was significantly negatively correlated with the N-Back (*r* = −0.5692, *p* = 0.0019), and the rDLPFC brain region was positively correlated with the N -Back reaction times was positively correlated (*r* = −0.6516, *p* = 0.0243), and LDLPFC brain region was positively correlated with N-Back (*r* = −0.5012, *p* = 0.0244).

**Conclusion:**

TMS improves cognitive function in post-stroke patients, changes in brain-derived neurotrophic factor concentrations under the influence of TMS, and also enhances connectivity in the bilateral DLPFC brain area network.

**Clinical trial registration:**

https://www.chictr.org.cn/showproj.html?proj=216761.

## Highlights

- TMS targeting the DLPFC is effective in enhancing cognition in poststroke cognitive impairment.
- Elevated BDNF and NGF is the important factors for TMS to promote cognitive function.
- TMS stimulate the DLPFC may improve cognitive functions by strengthening functional connectivity between brain networks.

## 1 Background

Globally, stroke is the second leading cause of death and the third leading cause of disability (1, 2). Cognitive impairment is a common complication after stroke with an incidence of 20–80% (3), and poststroke cognitive impairment (PSCI) remains highly prevalent and disabling (4). Studies have shown that approximately 40% of stroke patients suffer from dementia within a year after onset (5). The negative impact of PSCI is serious, but the rehabilitation process of conventional cognitive training is slow. A more efficient, safe, and low-cost cognitive rehabilitation method for PSCI is urgently needed.

Transcranial magnetic stimulation (TMS) is a non-invasive and relatively safe electrophysiological technique that utilizes pulsed magnetic fields to act on the central nervous system, transmitting magnetic signals through the cranial barrier without attenuation, stimulating the brain’s nerves and affecting their metabolism, blood flow, and neural oscillatory activity (6). In recent years, more and more studies have confirmed the efficacy of TMS in stroke (7). Evidence from previous studies suggests that high-frequency transcranial magnetic stimulation in the dorsolateral prefrontal area of the left prefrontal lobe (DLPFC) improves overall cognitive performance in elderly, cognitively impaired individual (8, 9). A randomized double-blind comparative trial conducted by Tsai et al. (10). showed that stimulation of the left DLPFC with high-frequency TMS was effective in modulating the release of dopaminergic neurotransmitters and improving cognitive-attentional function after stroke. Therefore, the DLPFC is often the target of choice for PSCI treatment, and the DLPFC is closely related to the cognitive control process, however, the specific mechanism of cognitive recovery after activating this brain region needs to be further explored.

Studies in the last decade have shown that brain-derived neurotrophic factor (BDNF) plays an important role in brain plasticity after ischemia in the central nervous system, and that increased concentrations of BDNF in the cerebral cortex may complete synaptogenesis and enhance dendritic spine formation and branching, which contributes to neuronal plasticity in stroke survivors (11, 12). BDNF is a member of the neurotrophic factor family and plays an important role in neuronal proliferation, survival and differentiation (13). NGF (14) is able to regulate neuronal structure and function, and has a role in regulating neuronal growth, development, and differentiation. rTMS magnetic field can stimulate phosphorylation, improve biological signaling response, promote neuronal cell growth, and regulate various types of neurotrophic factor (15). Although a few animal studies [e.g., (16)] have suggested that TMS may influence the expression of neurotrophic factors such as BDNF and NGF, there is still a lack of clinical research directly linking these changes to cognitive improvement in post-stroke patients.

Therefore, the present study considered the effect of TMS on cognitive function after stroke, further observed its changes on the concentration of brain-derived neurotrophic factor (BDNF) and nerve growth factor (NGF) in peripheral blood and assessed the functional connectivity of the brain region function and analyzed the correlation between neurotrophic factor, each brain region and clinical cognitive indexes. This study will provide a theoretical basis for the clinic.

## 2 Materials and methods

### 2.1 Trial design

This blinded randomized controlled trial was approved by the Ethics Committee of the First Hospital of Shanxi Medical University (No. KYLL-2023-264) and registered with the China Clinical Trial Registry (registration number: ChiCTR2400082383). Based on the results of the Montreal Cognitive Assessment (MoCA) in our preliminary trial, a sample size of 60 was planned at an alpha level of 0.05 to provide 90% power, assuming a loss to follow-up of 20%. We used random number generation software to assign subjects to group A (*n* = 30) and group B (*n* = 30). Patients in group A received TMS and patients in group B received sham stimulation.

**Figure 1:**
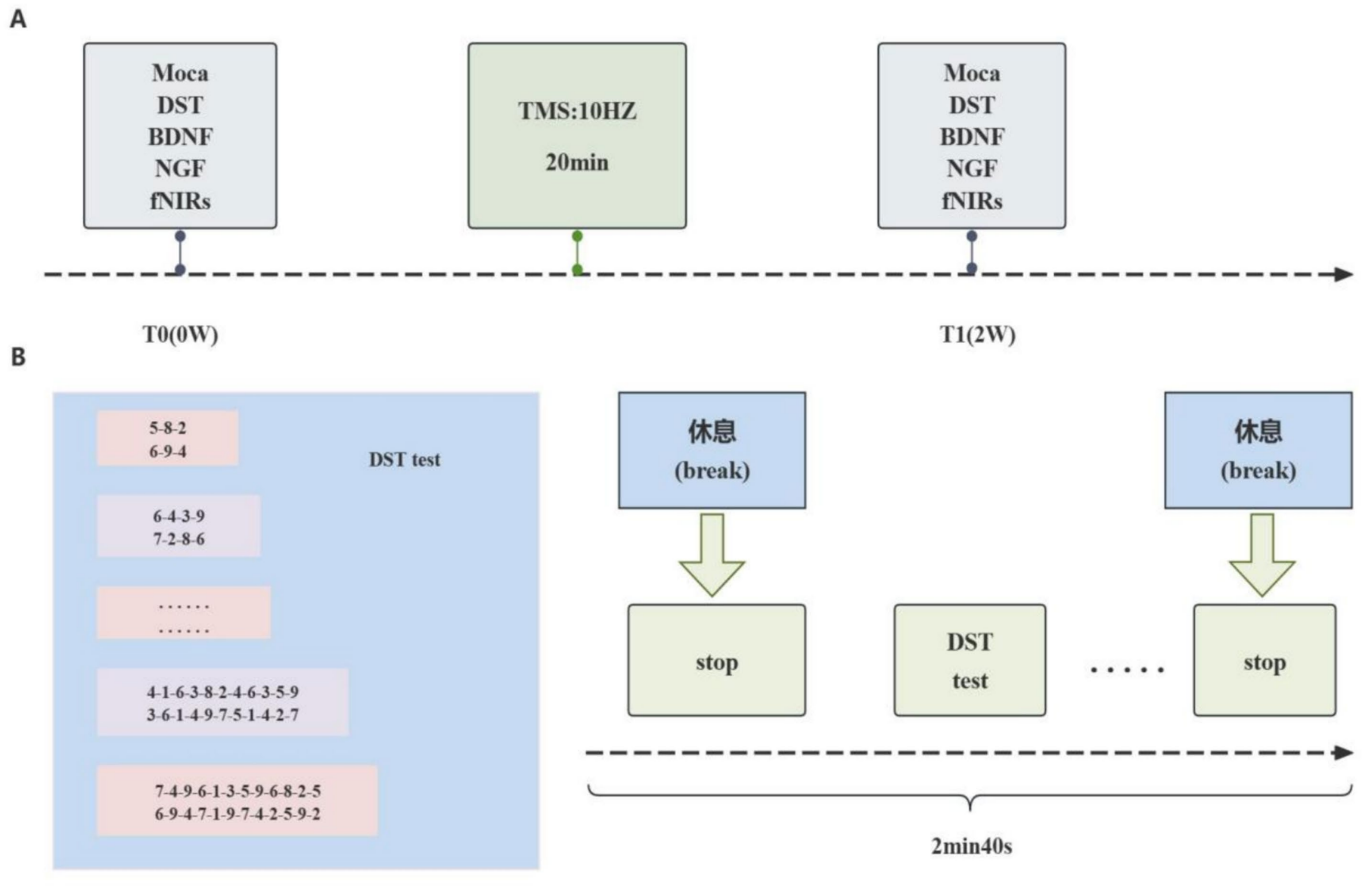
Study method. **(A)** The experimental process. Subjects were assessed for Montreal Cognitive Assessment (MoCA) and backward digit span test (DST) before (T0), and 2 weeks after (T1) transcranial magnetic stimulation (TMS), and for functional near-infrared spectroscopy (fNIRS) before and after treatment. Blood samples were collected from all patients prior to treatment for plasma detection. **(B)** Process of fNIRS acquisition and DST Test. fNIRS collection consisted of three blocks, each including a 60-s DST test and a 100-s break.

We selected 40 subjects willing and able to cooperate with the functional near infrared spectroscopy (fNIRS) assessment with a statistical power of at least 90% for the supplemental assessment from both groups receiving the physical therapy group. In addition to subject blinding, we blinded the scale assessor and the fNIRS collector. The process flow is shown in Figure 1.

### 2.2 Subjects

We recruited stroke patients from January 2024 to June 2024 in the Department of Rehabilitation Medicine of the First Hospital of Shanxi Medical University. The inclusion criteria were: (1) meeting the diagnostic criteria for stroke as confirmed by head CT or MRI; (2) stroke with hemiparesis for at least 2 weeks and not more than 6 months; (3) right-handedness on the Edinburgh Sharp Handedness Questionnaire; (4) aged 40–75 years old; (5) diagnosis of post-stroke cognitive impairment (PSCI) based on the 2023 Chinese Expert Consensus on Post-Stroke Cognitive Impairment, which requires a confirmed history of stroke, evidence of new-onset cognitive decline, and objective cognitive testing. In this study, we used MMSE ≤26 and/or MoCA ≤25 as cutoff scores to indicate cognitive impairment, in combination with clinical symptoms such as memory loss, reduced attention, or executive dysfunction; (6) no severe aphasia and able to complete cognitive tests; (7) stable vital signs and no progressive neurologic symptoms; (8) voluntary participation and signed informed consent.

Exclusion criteria were as follows: (1) complete left frontal lobe injury; (2) contraindications to TMS; (3) pre-stroke cognitive decline; and (4) severe neuropsychiatric and affective disorders affecting test results.

### 2.3 TMS interventions

The transcranial magnetic instrument produced by Wuhan Iridium was used. The patient was placed in the supine position or sitting position, the resting motor threshold (rMT) was measured by turning on the power, the electrode was placed on the small fusiform muscle of the left hand of the patient, and after the muscle was completely relaxed, a single TMS with a larger intensity was given to the M1 area innervating the muscle, and the waveforms and the stable motor evoked potentials (MEP) with a stable latency period were recorded, and the stimulation intensity was gradually reduced. After the motor evoked potential (MEP) was recorded, the stimulation intensity was gradually reduced, and the minimum intensity that could produce twitching of the left piriformis muscle in at least 5 out of 10 stimulations recorded by the EMG was the resting MT. The coil was tangential to the scalp during treatment, and the specific stimulation site was the left dorsolateral prefrontal cortex (DLPFC), the coil handle was backward, and the treatment was started after setting the stimulation parameters. Specific parameters: magnetic stimulation intensity of 80% of the resting motor threshold, frequency of 10 Hz, individual sequence stimulation time of 4 s, stimulation of 20 sequences, 20 min/treatment. Treatment was performed 7 times per week for 2 weeks.

### 2.4 Blood sample collection

Blood samples were collected from patients before treatment and after 2 weeks of treatment. Twenty-five milliliter of venous blood was collected via pre-venous puncture on the day of assessment from midnight to 08:00 am to 10:30 am in participants did not perform any exercise for blood sampling for 72 h. Twenty-five milliliter of venous blood was collected via pre-venous puncture on the day of assessment from midnight to 08:00 am to 10:30 am in participants who had fasted overnight. Participants did not perform any exercise for blood sampling for 72 h. Twenty-five milliliter of blood was collected and placed into serum separator tubes silicone tubes and plasma preparation tubes to allow blood to clot. The tubes were centrifuged at 3,500 relative centrifugal force for 10 min and aliquots of serum or plasma were collected without freeze–thaw cycles. In this study, we use the Human ELISA Kit from Wuhan Fearn Biologicals, and the concentrations of BDNF and NGF substances in blood samples were obtained by competitive ELISA in peripheral blood.

### 2.5 fNIRS

A 52-channel fNIRS system (ETG-4100, Hitachi Medical Systems, Japan) from the First Hospital of Shanxi Medical University was utilized for acquisition and preprocessing. The detection site mainly covered the prefrontal and bilateral temporal lobe regions, with 18 channels in the prefrontal lobe and 34 channels in the right and left temporal lobes. The sampling rate of the near-infrared spectra was 10 hz. fNIRS measurements consisted of a 30-s baseline, a 60-s task (DST), and a 70-s post-task recovery. Brain oxygenation variables measured by the fNIRS system were obtained (Figure 1). The entire experiment lasted approximately 2 min and 40 s while fNIRS data were acquired. Data were analyzed using Matlab R2017b (MathWorks, Natick, MA, USA). To facilitate subsequent functional connectivity (FC) and correlation analyses, we divided the 52 channels into 5 regions of interest (ROIs) based on the list of brain regions corresponding to the channels provided by the device development company. Changes in mean HBO in the left prefrontal lobe (LPF) were also observed, as well as differences in FC between all 5 ROIs. Detailed preprocessing steps and ROIs are shown in Supplementary Methods.

### 2.6 Assessment tools

This study assessed general cognitive functioning MoCA as well as changes in N-Back scores, Digit Breadth Test (DST), near infrared spectroscopy (fNIRS) oxidized hemoglobin (HBO). The scales were assessed before treatment (T0), and after 2 weeks of treatment (T1) by uniformly trained therapists who were not aware of the grouping. Pre- and post-treatment fNIRS data were collected and preprocessed by specialized collectors who did not know the subgroups.

### 2.7 Statistical analysis

SPSS 25.0 was used for statistical analysis. Measurement data were tested for normality using the Kolmogorov–Smirnov (K–S) test. Normally distributed data were expressed as mean ± standard deviation (X ± S), with paired-sample *t*-tests used for within-group comparisons and independent-sample *t*-tests for between-group comparisons. Non-normally distributed data were described as medians with interquartile ranges (P25, P75), and the Mann–Whitney U test was used. Categorical variables (e.g., gender) were compared using the chi-square test. A *p*-value of <0.05 was considered statistically significant.

For subjects with fNIRS data, a mixed-design repeated measures ANOVA was used to analyze differences in oxygenated hemoglobin (HBO) values and functional connectivity across five predefined regions of interest (ROIs) before and after the intervention. In this model, time (pre- vs. post-treatment) was treated as a within-subject factor, group (TMS vs. sham) as a between-subject factor, and age and sex were included as covariates to control for potential confounding effects. To address the issue of multiple comparisons across ROIs, Bonferroni correction was applied to control the family-wise error rate.

In addition, ANCOVA models were used to assess group differences in BDNF and NGF changes as well as cognitive outcome measures (MoCA, DST, N-Back), with age and sex included as covariates. Pearson correlation analyses were performed to examine associations between changes in neurotrophic factors and cognitive performance, as well as between fNIRS brain activation and behavioral outcomes. All tests were two-tailed, and statistical significance was set at *p* < 0.05, adjusted where necessary for multiple testing.

## 3 Result

### 3.1 Flow of participants through the trial

**Figure 2:**
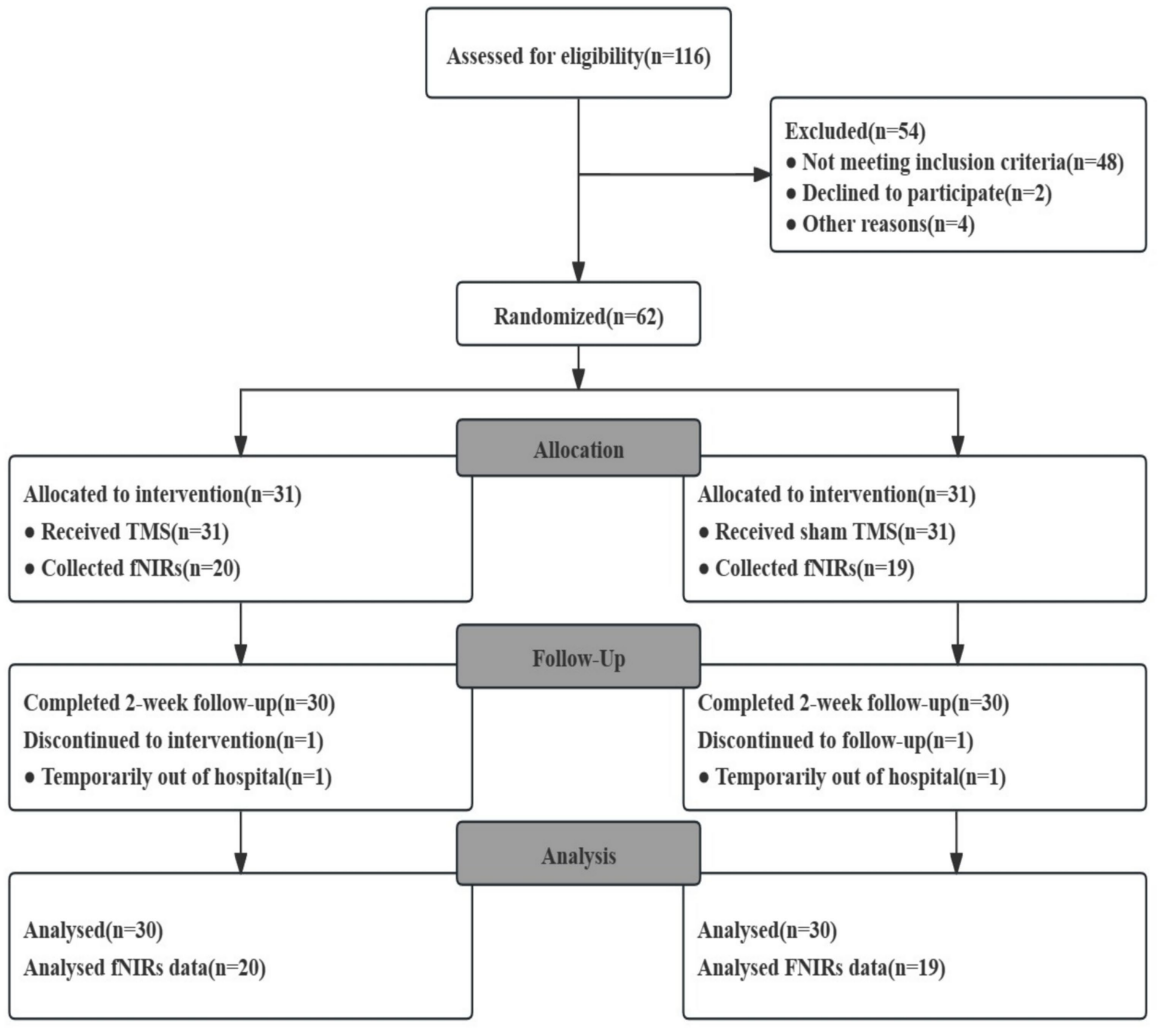
Flow diagram. There was no statistically significant difference between the two groups in terms of age, gender, disease duration, BDNF, NGF, DSA, MoCA, and N-Back baseline scores (*p* > 0.05).

Of the 116 patients enrolled, 62 completed baseline data collection and were randomized. Sixty patients completed the 2-week course, and 2 patients were discharged early for personal reasons. Sixty patients completed the entire 2-week follow-up and were ultimately included in the statistical analyses: group (1) (*n* = 30) and group (2) (*n* = 30). In addition, we randomly selected 40 patients who volunteered and were suitable for further evaluation and collected their fNIRS data. The flowchart is shown in Figure 2. The main clinical data of the 60 patients are shown in Table 1.

**Table 1:** Baseline participant characteristics.

Group	All patients			Patients with fNIRs		
	Group A (*n* = 30)	Group B (*n* = 30)	*p* value	Group A (*n* = 20)	Group B (*n* = 19)	*p* value
Sex [%]			0.599			0.650
Male	20 [66.7%]	18 [46.7%]		16 [80.0%]	14 [73.7%]	
Female	10 [33.4%]	12 [40.0%]		4 [50.0%]	5 [26.3%]	
Age	62.27	61.87	0.858	61.05	59.05	0.472
[95%CI]	[58.71 65.82]	[59.00 64.73]		[56.33 66.77]	[59.05 55.85]	
Course	13.63	13.03	0.538	12.40	12	0.674
[95%CI]	[12.23 15.03]	[11.63 14.44]		[11.06 13.74]	[10.55 13.45]	
BDNF	83.45 ± 45.02	75.74 ± 51.54	0.666	104.15 ± 41.38	97.77 ± 52.22	0.770
NGF	3.42 ± 1.35	2.87 ± 1.05	0.223	4.08 ± 1.15	3.39 ± 1.10	0.181
MoCA	18.20 ± 3.364	18.20 ± 3.364	1.000	20.10 ± 2.234	20.44 ± 2.068	0.732
DST	7.73 ± 0.961	7.00 ± 0.069	0.058	7.90 ± 1.11	7.44 ± 7.26	0.308
N-Back	1471.11 ± 228.97	1311.04 ± 236.60	0.070	1514.31 ± 190.39	1371.12 ± 257.47	0.183

### 3.2 TMS over the DLPFC to enhance cognition after stroke

**Figure 3:**
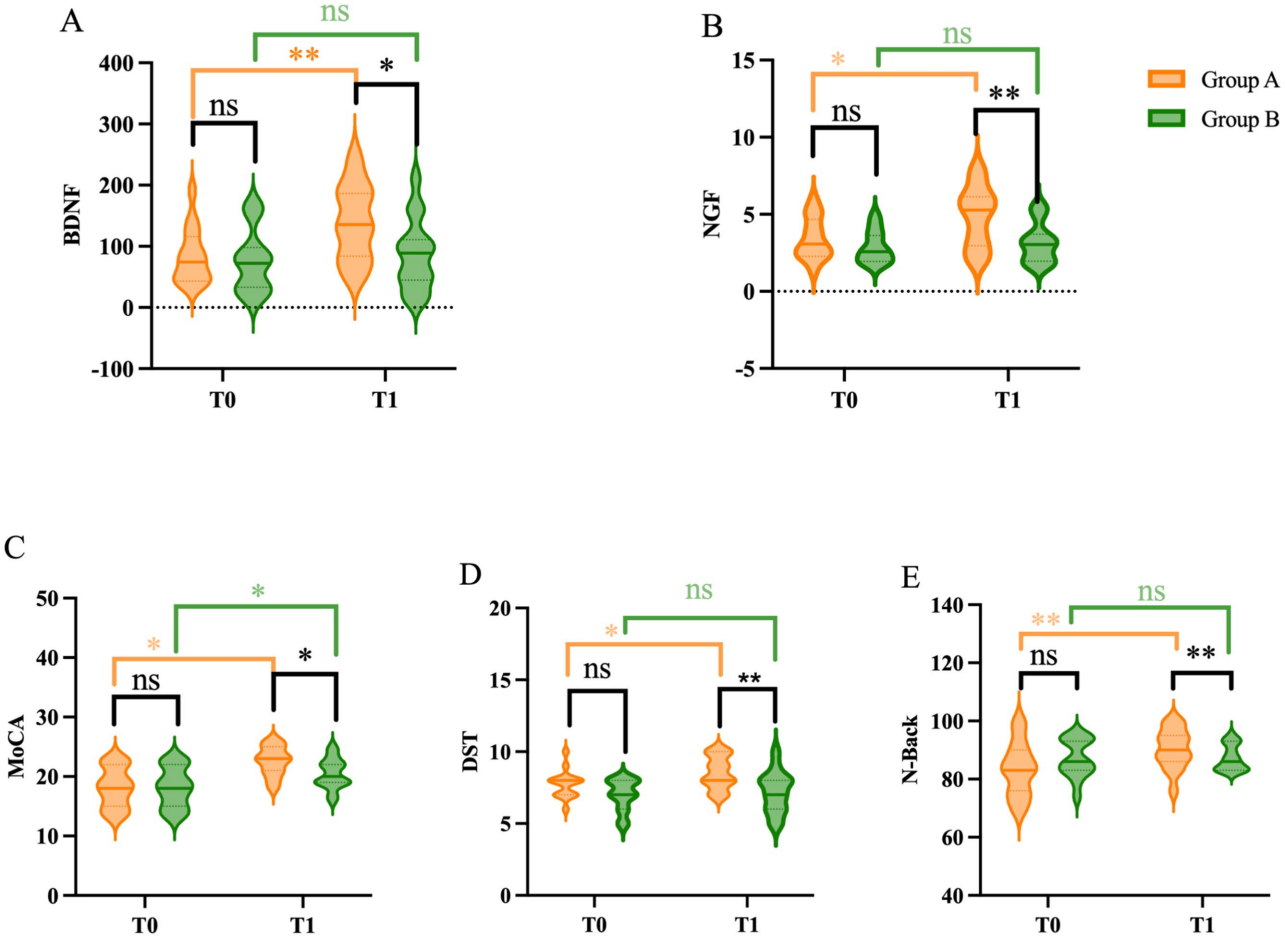
Changes in neuropsychological scales. **(A)** Peripheral blood BDNF concentration at baseline (T0), after 2 weeks of treatment. **(B)** Peripheral blood NGF concentration at baseline (T0), after 2 weeks of treatment. **(C)** Montreal Cognitive Assessment (MoCA) scores at baseline (T0), after 2 weeks of treatment (T1). **(D)** Digit span test (BDST) scores at T0 and T1. **(E)** N-Back scores at T0 and T1. *Indicates *p* < 0.05 for transcranial direct current stimulation Group A group compared with Group B; **indicates *p* < 0.01 for Group A group compared with Group B.

Following the 2-week intervention, patients in the TMS group (Group A) exhibited significant improvements in cognitive performance across multiple measures, Figure 3. Within-group comparisons revealed that MoCA scores improved significantly [*t* (29) = −2.388, *p* = 0.024], as did DST scores [*t* (29) = −2.093, *p* = 0.045]. N-Back reaction times also decreased significantly, indicating improved working memory speed [*t* (29) = 5.649, *p* < 0.001].

In contrast, the sham group (Group B) showed a modest improvement in MoCA scores [*t* (29) = −2.085, *p* = 0.046], while changes in DST [*t* (29) = −0.435, *p* = 0.667] and N-Back reaction times [*t* (29) = 1.624, *p* = 0.116] were not statistically significant.

Between-group comparisons at T1 showed that the TMS group achieved significantly higher MoCA [*t* (58) = −2.348, *p* = 0.026] and DST scores [*t* (58) = 2.845, *p* = 0.008], and significantly faster N-Back reaction times [*t* (58) = −3.038, *p* = 0.004] than the sham group. These results suggest that high-frequency TMS over the left DLPFC significantly enhances general cognitive function and working memory in post-stroke patients.

### 3.3 Changes in concentration of BDNF and NGF after TMS

The peripheral blood BDNF and NGF levels in both groups were elevated in T1 compared with T0, and the difference was statistically significant (*p* < 0.05), with BDNF (*t* = −2.845, *p* = 0.008) and NGF (*t* = −2.388, *p* = 0.024) in Group A; and BDNF (*t* = −0.513, *p* = 0.612), NGF (*t* = −0.631, *p* = 0.533).

The BDNF and NGF scores at T1 in both groups were significantly higher in Group A than in Group B. The difference was statistically significant (*p* < 0.05), with BDNF (*t* = 2.448, *p* = 0.021), NGF (*t* = 2.885, *p* = 0.007). The detailed description of the above data can be found in Table 2.

**Table 2:** Changes from T0 to T1 of MoCA in different linear mixed models.

Group	Group A (*n* = 30)				Group B (*n* = 30)			
	T0	T1	*t*	*p* value	T0	T1	*t*	*p* value
BDNF	75.75 ± 51.54	139.68 ± 61.92	−2.845	0.008^b^	80.89 ± 54.17	86.03 ± 58.02	−0.513	0.612
NGF	3.42 ± 1.35	4.89 ± 1.95	−2.388	0.024^a^	2.87 ± 1.05	3.14 ± 1.29	−0.631	0.533
MoCA	18.20 ± 3.36	22.60 ± 2.44	−2.388	0.024^a^	18.20 ± 3.36	20.47 ± 2.53	−2.085	0.046^a^
DST	7.73 ± 0.96	8.53 ± 1.13	−2.093	0.045^a^	7.00 ± 1.07	7.20 ± 1.42	−0.435	0.667
N-Back	1471.11 ± 228.97	1044.53 ± 181.96	5.649	<0.001^b^	1311.04 ± 236.60	1187.46 ± 175.79	1.624	1.116

The a represents *p* < 0.05; b represents *p* < 0.01. The student’t *t* test was used to analyze the results: 1. The difference between the BDNF, NGF, MoCA, DST, N-Back of Group A at 2 weeks after the start of the test (T1) and the BDNF, NGF, MoCA, DST, N-Back of Gourp B of T1 was statistically significant (BDNF: *t* = 2.448, *p* = 0.021^a^, NGF: *t* = 2.885, *p* = 0.007^b^, MoCA: *t* 2.348, *p* = 0.026^a^, DST: *t* = 2.845, *p* = 0.008^b^, N-Back: *t* = −0.416, *p* < 0.001)^b^.

### 3.4 TMS modulates the functional connectivity between the left and right prefrontal lobes by oxyhemoglobin

**Figure 4:**
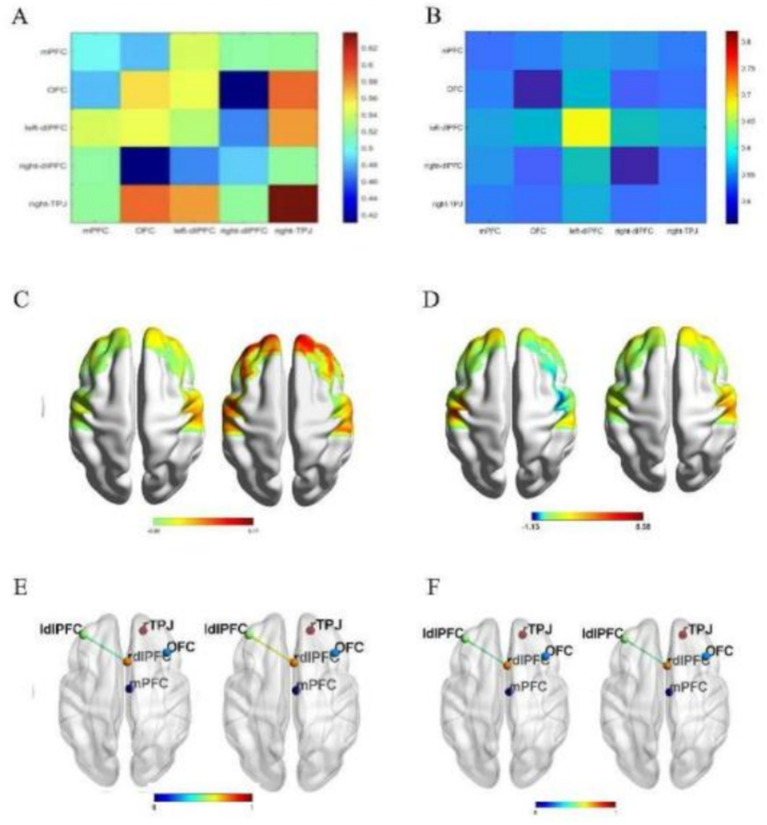
Changes in cerebral blood flow and functional connectivity of its brain regions. **(A)** The FC between ROIs after TMS stimulation. **(B)** The FC between ROIs after sham stimulation. **(C)** The functional connectivity (FC) between regions of interest (ROIs) after TMS stimulation. **(D)** The FC between ROIs after sham stimulation. **(E)** Changes in the mean oxygenated hemoglobin (HBO) level after TMS in the lDLPFC and rDLPFC. **(F)** Changes in the mean oxygenated hemoglobin (HBO) level after sham stimulation in the lDLPFC and rDLPFC.

The results showed that there was a temporal interaction effect of lDLFPC and rDLPFC functional connectivity strength in the TMS group after 10 stimulations, and the simple effects analysis showed that the before-and-after change in functional connectivity in the TMS group was significantly higher than that in the sham-stimulated group (*p* = 0.032). The functional connectivity main effects and interactions were not significant for the remaining brain regions. Functional connectivity between the left and right dorsolateral prefrontal cortex (DLPFC) increased in the TMS group after stimulation, while a decreasing trend was observed in the sham group. Notably, only the left DLPFC received TMS stimulation; the observed bilateral connectivity changes likely reflect interhemispheric network modulation induced by unilateral stimulation. (Figure 4).

### 3.5 BDNF, NGF and DLPFC brain regions after TMS and N-Back correlations

**Figure 5:**
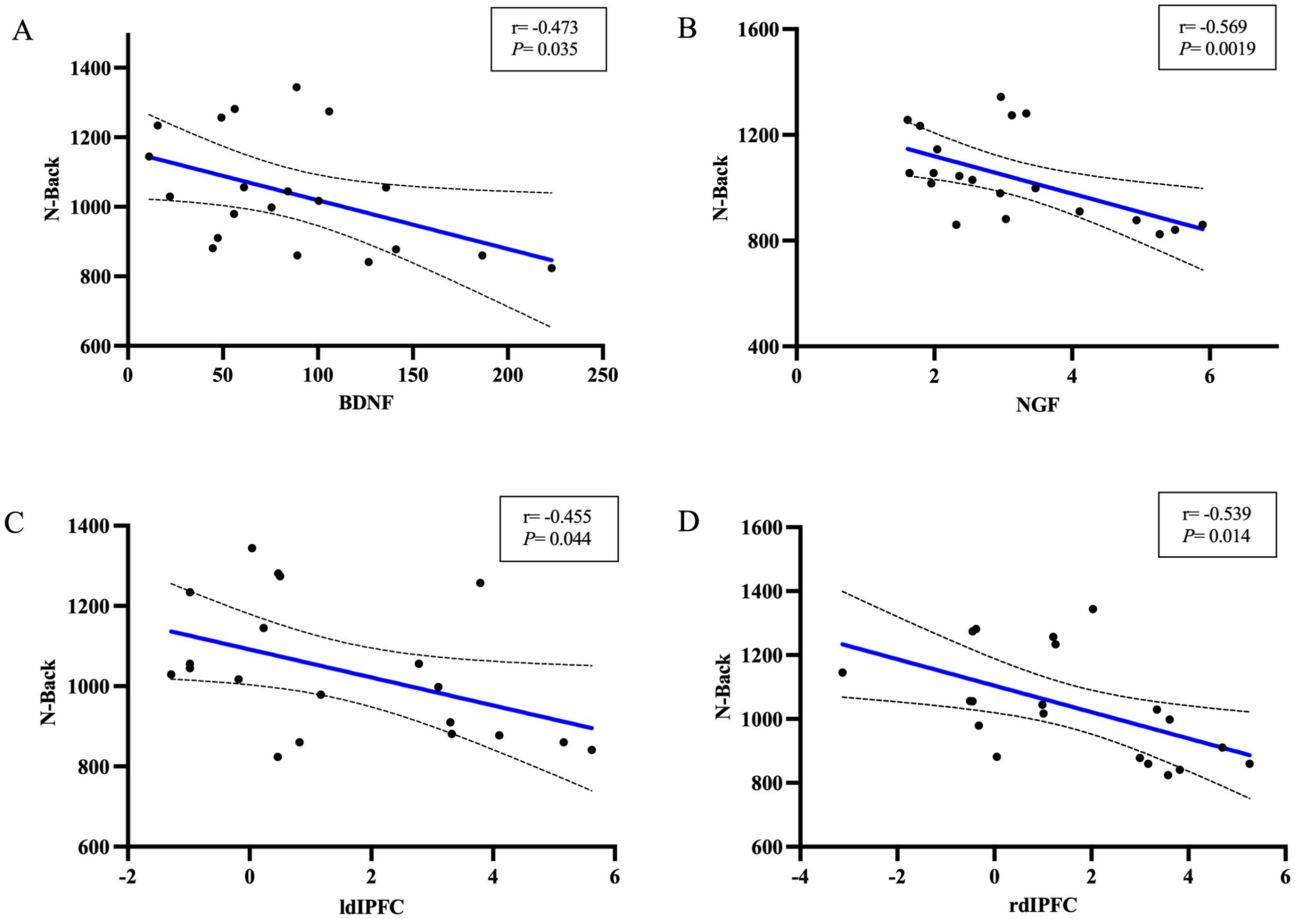
Correlation of neurotrophic factors and DLPFC regions with N-Back at T1. **(A)** Correlation of BDNF with N-Back. **(B)** Correlation of NGF with N-Back. **(C)** Correlation of lDLPFC with N-Back. **(D)** The correlation of rDLPFC with N-Back.

There was a correlation between plasma BDNF concentration at T1 in Group A at the time of 0-back response (*r* = −0.579, *p* = 0.023) as well as a significant negative correlation between NGF and the time of 0-back response (*r* = −0.717, *p* = 0.0026). As the concentration levels of BDNF and NGF increased, the N-Back reaction time was shortened, suggesting that the concentration levels of BDNF and NGF in Group A were closely related to cognitive function, As is shown in Figure 5.

The results showed that there was a correlation between the left frontal blood oxygen change values and N-Back reaction time at T1 in the TMS group (*r* = 0.5042, *p* = 0.0329) as well as in the right frontal lobe as well (*r* = 0.5429, *p* = 0.0243). It is suggested that the values of blood sample changes in bilateral frontal regions after stroke may be closely related to cognitive function.

## 4 Discussion

In this study, 60 patients with PSCI were recruited as participants, and the left DLPFC improved MoCA, DST, and N-Back scores, increased peripheral blood BDNF and NGF concentrations, and correlated with the N-Back after 2 weeks of TMS (10 Hz) stimulation, suggesting that TMS had a major impact on the improvement of general cognitive functioning, which may be due to the shortened patients’ reaction time and interaction with working memory aspects. We also found that TMS modulated changes in HBO content in the brain, activated the DLPFC brain region, promoted functional connectivity in the bilateral DLPFC, and correlated positively with N-Back reaction time, implying that with the activation of the bilateral DLPFC brain region, the patients’ reaction time to do the task was significantly shortened, which may have had a beneficial effect on their cognitive function. This study demonstrated that TMS can improve patients’ cognitive functions as shown by the imaging results of fNIRS, which can better regulate brain plasticity and thus promote the reorganization of neural brain networks.

### 4.1 Effect of TMS on cognition in PSCI patients

In recent years, a large number of studies have used TMS for the treatment of PSCI, all of which have achieved more satisfactory result (17), which is basically consistent with our results. With the deepening of the studies, the controversies over the frequency of TMS and the site of stimulation have been narrowed down to a certain extent (18, 19). In terms of the site of stimulation, most TMS studies have placed the coil at the scalp tangential site of the left DLPFC (20, 21), a key center for executive function, working memory, and reasoning (22). A recent meta-analysis covering 19 studies showed that stimulation of the right hemisphere DLPFC was effective in improving overall cognition in patients with PSCI, but TMS stimulation of the left hemisphere DLPFC was a more effective area of stimulation and significantly improved overall cognitive functioning, memory, attention, and executive functioning in patients with PSCI (23). Cognitive recovery is slow and may not cause significant changes in behavioral performance in a short period of time. However, the present study showed that the MoCA, DST, and N-Back scores of PSCI patients improved after stimulation in the TMS group compared to the sham-stimulation group, suggesting that TMS may have affected cognitive functions in the brain within a short period of time, which can be detected by brain imaging as well as peripheral blood neurotrophic factor.

Interestingly, a modest improvement in MoCA scores was also observed in the sham stimulation group (Group B), despite no significant changes in DST or N-Back performance. This may be explained by several factors. First, spontaneous cognitive recovery is known to occur in some post-stroke patients, particularly within the subacute phase. Second, a practice effect cannot be ruled out, as repeated exposure to the MoCA test over a short interval can sometimes result in score increases. Additionally, non-specific effects such as patient engagement, attention from clinical staff, or expectations of benefit (i.e., placebo effect) may also have contributed to improved cognitive performance. These factors highlight the importance of including objective neurophysiological and neurochemical measures, such as fNIRS and neurotrophic factor levels, to complement behavioral outcomes.

### 4.2 Effect of TMS on peripheral blood BDNF and NGF concentrations

Post-stroke cognitive dysfunction is mainly manifested as a decline in executive tasks, and the decline in executive function is closely related to the prefrontal lobe. Brain-derived neurotrophic factors, BDNF and NGF, are mostly distributed in the prefrontal region and hippocampal region of the brain (24). NGF and BDNF are intrinsic neurotrophic factors that exert neuroprotective effects after mature brain injury. The neurotrophic factors BDNF and nerve growth factor (NGF) are key mediators of neuronal plasticity, neuronal survival, and functional recovery by binding to the pro-myosin receptor kinase (Trk) receptor (25, 26) Cellular and molecular studies have demonstrated that the neurotrophic factor BDNF, NGF, plays an important role in regulating the functional dynamics of executive function and memory-related brain regions (27, 28). After stroke, decreased levels of BDNF affect the regulation of neuroplasticity and the potential for self-repair (10). NGF is beneficial in ameliorating symptoms of neurological deficits and promoting neurological recovery (29). Recent studies have suggested that external magnetic fields induced by TMS may affect BDNF levels in serum and cerebrospinal fluid (CSF) (30).

TMS can be used to regulate neuroplasticity (31–33). In this study, we investigated the effect of TMS treatment for PSCI in relation to BDNF and NGF. The results showed that after 2 weeks of treatment, serum BDNF and NGF levels were significantly higher in the TMS group than in the sham stimulation group. This is consistent with the results of a previous study, which found that TMS treatment reduced glioblastogenesis and promoted the expression of BDNF and NGF in the peri-infarct area after stroke (16).

TMS magnetic field can stimulate phosphorylation and better improve the biological signal response, promote the growth of nerve cells, and regulate various types of neurotrophic factors (15), which explains the higher levels of BDNF and NGF in this study’s TMS group than in the sham stimulation group.

In addition the results of the present study further Pearson correlation analysis showed that there was a significant inverse relationship between the increased values of serum BDNF and NGF and the N-Back response time, which is similar to the results of Butterfield’s study showing a significant correlation between oxidative damage and the progression of neurodegenerative diseases (34). It is hypothesized that TMS may have improved cognitive function by decreasing N-Back response time through upregulation of BDNF and NGF levels.

Taken together, our findings suggest that TMS treatment may have potential therapeutic effects on PSCI patients by upregulating BDNF and NGF expression to enhance neuroplasticity and neuroprotection, and to promote cognitive recovery. Here, it is noteworthy to mention that changes of candidate biomarkers reported in our study might help in the future in adopting a more tailored approach to treat PSCI patients with rTMS (35–39).

### 4.3 TMS regulates oxygenated hemoglobin and functional connectivity of brain regions

The pathogenesis of PSCI, a vascular cognitive dysfunction, is unclear, and changes in cerebral blood flow have been considered (40). Shang et al. (41) applied 20 Hz TMS to the dorsolateral aspect of the left prefrontal lobe, and showed that there was a redistribution of cerebral blood flow, with an increase in the relative blood flow in the left medial temporal cortex and hippocampus, and a decrease in the precuneus and cerebellar regions. It has also been stated that no significant relationship between increased cerebral blood flow and cognition was observed (42). It can be seen that the conclusion that TMS causes changes in blood flow and thus improves cognitive performance is not uniform and still needs to be further explored.

TMS stimulation of DLPFC sites can enhance cerebral blood perfusion and modulate neurotransmitter transmission in the brain through neurovascular coupling, which can help to restore functional homeostasis and exert cerebral neuroprotection in bilateral DLPFCs in the brain, thereby altering synaptic plasticity (43). TMS-induced changes in regional activity have been shown to propagate to interconnected brain regions, thereby affecting the entire network of stimulated nodes within the network of activity (44), which is consistent with our findings. However, these mechanisms have rarely been demonstrated in clinical studies.

fNIRS is well suited as a method to monitor the transient and long-term effects of transcranial magnetic stimulation (45). The fNIRS results in this study showed a significant increase in local cerebral blood perfusion in the left prefrontal lobe, and a significant increase in functional connectivity between the left prefrontal lobe and the right prefrontal lobe, which may imply an increased connectivity of bilateral frontal lobe networks, and thus we can assume that TMS showed its remote effects, suggesting that TMS may have had an impact on brain function, improving the state of the patient’s cerebral network connectivity and promoting the reorganization of neurobrain networks and shaping post-stroke brain networks, thereby improving cognitive function in PSCI patients. This is consistent with the findings of Selingardi et al. (46), who also found that transcranial magnetic stimulation promotes localized neural regeneration, enhances neuroplasticity and inter-cortical connectivity, and displays the remote effect of TMS. We also found that LDLPFC and RDLPFC brain regions correlated with N-Back, suggesting that TMS may have improved cognitive function by activating working memory.

However, the brain regions in this study did not show significant correlations with MoCA scores. Although MoCA scores improved significantly in the TMS group, the magnitude of improvement was relatively smaller and more variable compared to task-based measures such as the N-Back. This reduced sensitivity may be due to the multidimensional nature of the MoCA, which evaluates several cognitive domains broadly but may not capture subtle changes in specific neural circuits or functions targeted by the intervention. In addition, the modest sample size (approximately 30 subjects per group) likely limited the statistical power to detect associations between fNIRS signals and global cognitive outcomes. Taken together, these findings suggest that while MoCA can reflect overall cognitive trends, it may not be the most sensitive correlate of localized prefrontal activation. Future studies with larger cohorts and more granular cognitive assessments are warranted to clarify the relationship between neuroimaging biomarkers and global cognitive performance. Given its sensitivity to regional hemodynamic changes, fNIRS remains a promising tool for monitoring neuroplastic responses to interventions such as TMS.

### 4.4 Limitations

There are some limitations to this study. First, event-related potentials (ERPs) and electroencephalograms (EEGs) were not collected because it was difficult for PSCI patients to cooperate in the collection of ERPs and EEGs; future studies should combine EEGs, ERPs, and fNIRS data to obtain results with high temporal and spatial resolution. Second, the sample size of fNIRS data is small. The sample size of the data should be expanded in the future when conditions permit. Third, we did not study the sustained effect of TMS intervention, so in the future, we should follow up the patients’ efficacy 1 month and 6 months after treatment.

## 5 Conclusion

TMS treatment of the DLPFC region may improve cognitive functions by strengthening functional connectivity between brain networks and improving intracerebral metabolism, increasing cortical excitability. Elevated BDNF and NGF in peripheral blood is one of the important factors for TMS to promote cognitive function recovery in ischemic stroke patients. Therefore task state fNIRS combined with the expression of BDNF and NGF factors in peripheral blood can be considered as a promising biomarker of cognitive deficits after stroke, which can predict the therapeutic response for the modulation of functional brain networks in PSCI.

## Data Availability

The raw data supporting the conclusions of this article will be made available by the authors, without undue reservation.
